# Dissecting the Non-human Primate Brain in Stereotaxic Space

**DOI:** 10.3791/1259

**Published:** 2009-07-16

**Authors:** Mark W. Burke, Shahin Zangenehpour, Denis Boire, Maurice Ptito

**Affiliations:** Department of Physiology, Universite de Montreal - University of Montreal; School of Optometry, Universite de Montreal - University of Montreal; Département de chimie-biologie , Université du Québes à Trois-Rivières

## Abstract

The use of non-human primates provides an excellent translational model for our understanding of developmental and aging processes in humans^1-6^. In addition, the use of non-human primates has recently afforded the opportunity to naturally model complex psychiatric disorders such as alcohol abuse^7^. Here we describe a technique for blocking the brain in the coronal plane of the vervet monkey (*Chlorocebus aethiops sabeus*) in the intact skull in stereotaxic space. The method described here provides a standard plane of section between blocks and subjects and minimizes partial sections between blocks. Sectioning a block of tissue in the coronal plane also facilitates the delineation of an area of interest. This method provides manageable sized blocks since a single hemisphere of the vervet monkey yields more than 1200 sections when slicing at 50μm. Furthermore by blocking the brain into 1cm blocks, it facilitates penetration of sucrose for cyroprotection and allows the block to be sliced on a standard cryostat.

**Figure:**
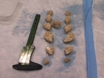


## Protocol

### Part 1: Pre-processing of tissue

Tissue should be well perfused with paraformaldehyde, glutaraldehyde, or formalin. This can be achieved through standard transcardial perfusion typically used to harvest other organs. In the present study the subject was deeply sedated with ketamine hydrochloride (10 mg/kg, i.m.), euthanized with an overdose of sodium pentobarbital (25 mg/kg, i.v.) and perfused transcardially with 0.1 M PBS until completely exsanguinated.This is followed by a 4% paraformaldehyde solution in PBS for 5 min (~1 liter).

### Part 2: Stereotaxic blocking

Prior to placing the head into the stereotaxic frame the coordinates of the Horsley-Clarke/interaural plane zero needs to be taken. This is the theoretical midpoint between the ears. To measure this plane, the ear bars need to be equally fitted in the apparatus, then place a scalpel blade in the stereotaxic manipulator arm and measure the midpoint between the ear bars. This is important for determining where to block the tissue based on research needs. Once this is completed the skull needs to be prepared for fixation into the stereotaxic apparatus.
In order to place the head into the stereotaxic frame the lower jaw will have to be removed with bone rongeurs and a scalpel. Additionally the skin, muscle, and connective tissue should be removed in order to expose the skull. Once the connective tissue is removed from the skull, expose the brain by chipping away the calvaria (the tabular portion of the occipital bone as well as the parietal, ad frontal bones). In the present experiment part of the calvaria has all ready been removed. Be careful not to completely remove the temporal bones because the ear canals will need to be intact to place the head into the stereotaxic frame. The remaining dura matter should be removed from the exposed brain. The skull is now ready to be placed into the stereotaxic frame.
In the same manner in which one would perform a stereotaxic surgery, adjust the eye, palate, and ear bars such that the head is securely fixed in the stereotaxic apparatus. Place the stereotaxic manipulator in the predetermined anterior/posterior (A/P) coordinates and move the manipulator to the lateral aspect of the brain. Slowly lower the blade into the brain, completely raise the blade from the brain, then move medially and lower the blade again. Repeat these two steps until the blade has reached the lateral aspect of the opposite hemisphere. This completes the first coronal block. For subsequent coronal blocks move the manipulator 1cm in the A/P axis and repeat until the entire brain has been blocked.

### Part 3: Removing the brain from the skull

Remove the head from the stereotaxic frame and hold the exposed brain in the palm of your hand. Try to secure the brain by lightly placing coddling the brain in the palm of your hand and place your pinky finger across the frontal lobes, this minimizes movement of the brain in the skull. In order to prevent the drying of the pial surface of the brain, place a piece of PBS dampened gauze across the brain. Hold the head firmly by the skull and chip away the remaining occipital and temporal bones along with the spinal column. This exposes the base and lateral aspects of the brain. Lastly remove any remaining frontal bone and the nasal bone, which will allow access to the olfactory bulbs. It is important to remove the frontal bone last because as you remove the base of the skull the brain moves slightly and the frontal lobes can become damaged on the jagged edges of the fontal bone. Cut away and remove remaining dura matter. Carefully lift the front of the brain, slide the scalpel under the brain and free the brain from the skull.

### Part 4: Measurements

There are a number of useful measurements that can be made prior to freezing. For example, the A/P axis of the brain with a caliper. Furthermore the specific density can be measured by weighing the brain, measuring volume by the displacement of water in a graduated cylinder then dividing weight by volume displacement (Table 1).

### Part 5: Finish blocking the brain.

Typically when the scalpel blade is inserted into the brain it is not long enough to completely penetrate the full dorsal-ventral extent of the brain. Once the brain is removed and gross measurements are obtained, take a tissue-slicing blade and finish blocking the brain through to the ventral side of the brain (Figure 2).
Prior to freezing the blocks, it is necessary to cryoprotect the tissue in graded PBS-buffered sucrose solutions (10, 20, and 30%) maintained at 4˚C until the brain sinks. It typically takes an overnight incubation in 10%, 2-3 days in 20% and additional 3-5 days in 30% for the blocks to sink to the bottom of the container. A daily change of the 30% sucrose solution is recommended.

### Part 6: Representative Results

There are a number of gross morphological measurements that can be made once the brain has been removed from the skull. These include A/P length, weight, and specific density (Table 1). We generally block the brain into 6-7 blocks measuring 1cm (Figure 1). Each piece is then photographed (Figure 2) and can be further dissected depending on research needs or prepared for freezing in graded sucrose solutions.

**Table:** 

**Subject**	**A/P Length(mm)**	**Weight(grams)**	**Water Displacement(ml)**	**Specific Density(g/ml)**
O2303-2-1-1	64.3	28.1	24	1.171
O5180-1	71.3	38.7	34	1.138
O2708-3-1	62.8	28.7	26	1.104
O9184-4-2	65.3	29.5	24	1.229
N459-1-14-2	68.2	31.6	26	1.215
AVERAGE	66.38	31.32	26.8	1.171
STD DEV	3.38	4.33	4.17	0.052

**Table 1. **Gross Morphological Measurements of the Right Hemisphere of 5 Month Old Vervets


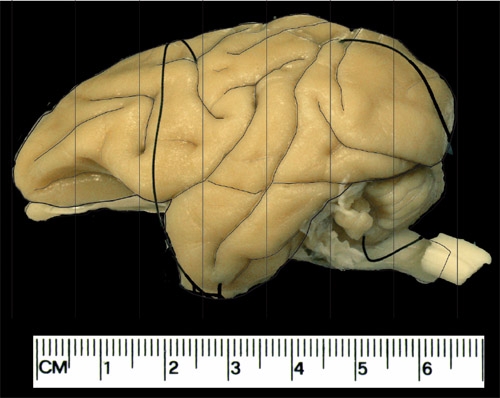
 
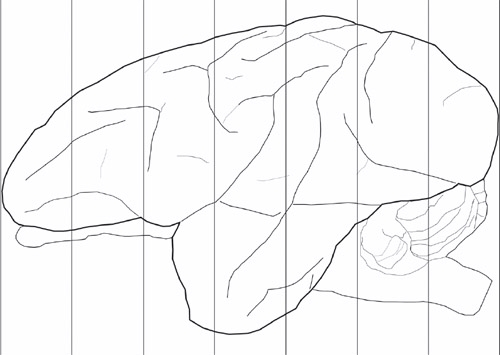
 **Figure 1. Schematics for the coronal planes used for blocking the brain.** This is an example of an externalized adult vervet brain. Example of the blocking procedure. The vertical lines here are spaced at 1cm, typically producing 7 coronal blocks from each hemisphere.


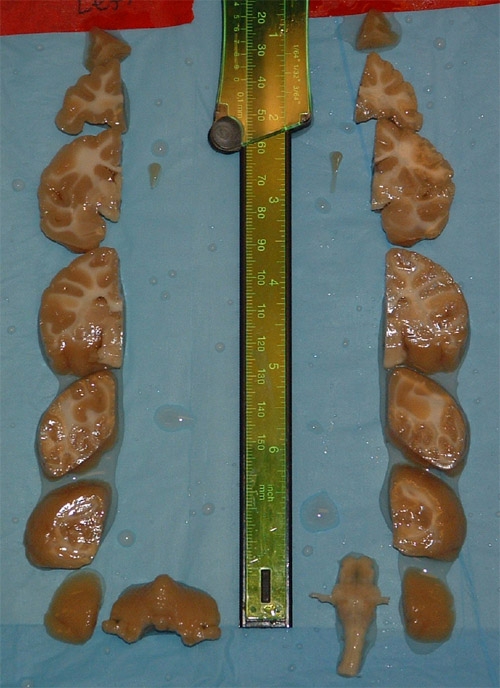
 **Figure 2. Blocks of brain tissue in stereotaxic space.**Each block will yield approximately 200 sections taken at 50µm. With this sampling scheme over 1200 sections through the cortex will be taken and an additional 400-500 from the cerebellum when sliced in the coronal plane.

## Discussion

The St. Kitts vervet (*Chlorocebus aethiops sabeus*) is an Old World primate with similar patterns and rates of cortical and subcortical brain development to that of humans. This species has been used to model complex human behavioral disorders like anxious behavior, hypertension^8^, hemispherectomy^9^, Parkinson’s disease^10^, Alzhemier’s disease^11^, and alcohol abuse^12^. More recently, this species has been used to study the neuroanatomical effects of naturalistic prenatal ethanol exposure. Pregnant vervets were allowed to drink the equivalent of 3-5 standard drinks four times a week during the third trimester and we report that there is a 35% reduction of neurons in the frontal cortex^13^. The brains for this study were stereotaxically blocked such that only 3 of 7 blocks needed to be sectioned to complete the stereological evaluation of the frontal cortex. This particular technique is not limited to the non-human primate brain, but can be extended to rodent brains as well. For example, if the somatosensory cortex of a rat is the region of interest, a stereotaxic cut anterior and posterior to that region can be made using the rodent stereotaxic frame. This provides a small region to section and a standard coronal plane between animals. It is important to note that the blade must be fully retracted from the brain before any medial-lateral movement is made with the stereotaxic manipulator otherwise the blade will un-necessarily damage the brain.
